# Characterisation of porin genes from *Mycobacterium fortuitum *and their impact on growth

**DOI:** 10.1186/1471-2180-9-31

**Published:** 2009-02-09

**Authors:** Soroush Sharbati, Kira Schramm, Sonja Rempel, Hwa Wang, Ronny Andrich, Verena Tykiel, Ralph Kunisch, Astrid Lewin

**Affiliations:** 1 Freie Universität Berlin, Institute of Veterinary Biochemistry, Oertzenweg 19b, 14163 Berlin, Germany; 2 Robert Koch-Institute, Nordufer 20, 13533 Berlin, Germany; 3 Deutsches Institut für Bautechnik (DIBt) Section II 4 – Health and Environmental Protection, Kolonnenstr. 30 L, 10829 Berlin, Germany

## Abstract

**Background:**

Highly pathogenic mycobacteria like *Mycobacterium tuberculosis *are characterised by their slow growth and their ability to reside and multiply in the very hostile phagosomal environment and a correlation between the growth rate of mycobacteria and their pathogenicity has been hypothesised. Here, porin genes from *M. fortuitum *were cloned and characterised to address their impact on the growth rate of fast-growing and pathogenic mycobacteria.

**Results:**

Two genes encoding porins orthologous to MspA from *M. smegmatis, porM1 *and *porM2*, were cloned from *M. fortuitum *strains, which were originally isolated from human patients. Both porin genes were at least partially able to complement the mutations of a *M. smegmatis *mutant strain lacking the genes *mspA *and *mspC *with respect to the growth rate. *PorM1 *and *porM2 *were present in different strains of *M. fortuitum *including the type strain. Comparative expression analysis of *porM *genes revealed divergent porin expression among analysed *M. fortuitum *strains. Repression of the expression of porins by antisense technique decreased the growth rates of different *M. fortuitum*. The effects of over-expression of *porM1 *as well as *porM2 *varied depending on the strain and the concentration of antibiotic added to the medium and indicated that PorM1 and PorM2 enhance the growth of *M. fortuitum *strains, but also the diffusion of the antibiotic kanamycin into the cells.

**Conclusion:**

This study demonstrates the important role of porin expression in growth as well as antibiotic susceptibility of the opportunistic bacterium *M. fortuitum*.

## Background

*Mycobacterium *is considered a diverse genus with highly pathogenic members like *M. tuberculosis *or *M. leprae *as well as less pathogenic, opportunistic and saprophytic species belonging to the so-called rapidly growing mycobacteria (RGM). The species of RGM able to cause human disease basically belong to the *M. fortuitum *group, the *M. chelonae/abscessus *group and the *M. smegmatis *group. Members of these groups are commonly seen in aquatic environments like municipal tap water, and health care-associated outbreaks are often associated with contact to tap water or water sources such as ice.

The *M. fortuitum *group includes three taxa: *M. fortuitum*, *M. peregrinum *and a third biovariant complex. The *M. fortuitum *group is involved in 60% of localised cutaneous infections in immunocompetent persons caused by RGM but is a rare cause of pulmonary disease. Most or all of the cases of community-acquired or health care-associated diseases caused by the *M. fortuitum *group are due to *M. fortuitum*. This species basically causes skin lesions, wound infections, postinjection abscesses, postsurgical wound infections or pulmonary disease in previously healthy hosts [1]. Little is known about the virulence mechanisms and persistence of this human pathogen. However, Cirillo *et al*. [2] and Da Silva *et al. *[3] reported that *M. fortuitum *was capable to replicate in amoebae and murine monocytic cells, respectively.

In a previous study, we showed that the intracellular survival of *M. smegmatis *depended on the amount of porins in the mycobacterial outer membrane (OM). The mutant strain ML10 of *M. smegmatis*, which lacks the porins MspA and MspC [4], exhibited significantly enhanced intracellular survival compared to the parental strain SMR5 [5]. MspA belongs to a novel class of mycobacterial OM proteins present in many RGM but apparently absent in slowly growing mycobacteria [6]. The main porin of *M. smegmatis*, MspA, is an extremely stable octameric protein composed of 20 kDa monomers [7] and provides the uptake of hydrophilic nutrients across the extraordinarily restricting mycobacterial OM [7,8]. By means of DNA hybridisations using a probe derived from the *mspA *sequence, Niederweis and colleagues [6] indicated that the genome of *M. fortuitum *contained orthologous porin genes.

Since the saprophytic bacterium *M. smegmatis *causes disease only in rare cases [1] and shows a very limited intracellular persistence [5], it is important to investigate the role of porins on virulence in pathogenic members of RGM, which are able to multiply intracellularly. *M. fortuitum *was suggested to be a suitable model *Mycobacterium *[9]. Like *M. tuberculosis*, it resides intracellularly in vacuoles restricting interferon-γ-induced nitric oxide production and limits the maturation of phagosomes [3]. Therefore, *M. fortuitum *was chosen to detect and characterise porins and to analyse their impact first on extracellular growth and in a later stage on intracellular growth. For this purpose, we used two different *M. fortuitum *strains (10851/03 and 10860/03) that originally were isolated from human patients. Comparative analysis was performed using also the type strain *M. fortuitum *DSM 46621.

## Results

In order to verify the taxonomic classification and to define the phylogenetic relationship between the strains analysed, complete sequences of the 16S rRNA genes were determined using the primers, which were described by Adekambi and Drancourt [10]. The phylogenetic analysis of the 16S rRNA sequences confirmed the taxonomic classification of the *M. fortuitum *strains employed (data not shown).

**Figure 1:**
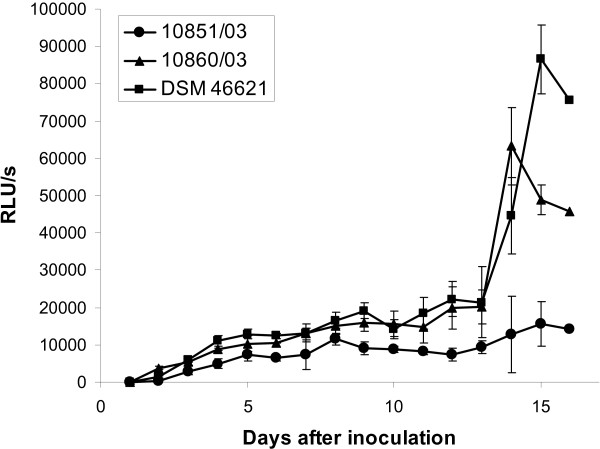
**Growth rate of the *M. fortuitum *strains 10851/03, 10860/03 and DSM 46621**. The growth rate of the strains was measured by quantification of the ATP-content [displayed as relative light units (RLU)] in broth cultures.

Comparison of growth rates of the employed strains was performed in broth by measuring the ATP content of the cultures. Compared to other methods for growth measurements such as OD-measurement, cfu-counting or quantification of DNA, the quantification of the ATP-content has the advantage of not being biased by clumping of cultures or by inability of viable bacteria to grow on agar if plated from a broth culture or by occurrence of dead bacteria. We therefore chose this method for the comparison of the growth rates of the three strains. However, the ATP content of bacteria may vary depending on their physiological state and it therefore has to be considered as a surrogate growth marker. As shown in Figure 1 (also see Additional file 1), strain 10851/03 only grew very poorly, while strains 10860/03 and DSM 46621 multiplied strongly from day ten until day 14 or until day 15, respectively.

### *PorM *genes of *M. fortuitum *are orthologs of *mspA*

To detect porin genes orthologous to *mspA *in *M. fortuitum*, preliminary hybridisation experiments were performed with a probe derived from the main porin gene of *M. smegmatis mspA *(accession no.: AJ001442) using the primers hpor and npor (Table 1) covering nucleotide 8 to 697. The probe hybridised to the genomic DNA from *M. fortuitum *strains (data not shown). Thus, orthologs seem to exist in all strains analysed.

**Table 1:** Primers and probes used in this study.

Gene	Primers or probes	Sequence 5'-3'
*mspA*	hpor	5'-CGG TCT CAG CGA CCG AAC-3'
	npor	5'-CCG GCG ATA CAG TTA GGG A-3'

*porM1*	mf-4IV-fw	5'-TCT CCA GGG GCT GCT TTT G-3'
	mf-4-bw	5'-CGG GAC GCC AAC CAC ATA AC-3'

*porM1*	komf-3f	5'-CTG AAG CTT CAC CGA GCT GAG CAT CCT CAC-3'
	komf-4b	5'-GAC ACT AGT CGT TGG CTA CAG AAC AAC ATT CC-3'

*porM2*	porM2-51-fw	5'-ATG AAG GCA TTC AGT CGG G-3'
	porM2-51-bw	5'-TGC TCC TCA AAG GAG AAG CG-3'

*porM2*	porM2-rev-1	5'-CGC TTC TCC TTT GAG GAG CA-3'
	porM2-rev-2	5'-TCC AGA CCC GCA TGA GAT ACG-3

*porM2*	porM2-fw-hind	5'-ACA AGC TTC AGC AAC GCT GTG AAC GCA-3'
	porM2-bw-hpa	5'-CAG TTA ACA CTA CGG GAC GCT CGT GTC C-3'

*porM2*	porM2-rna-fw	5'-CGC AAG CCT CTT CGT CGG C-3'
	porM2-rna-bw	5'-CCA AGG TGC CCT CGA ACT CAT C-3'

*porM1*	porM1-as-1	5'-CGG ATC CTA GGG AGA ACA TGA AGG CAT TCA G-3'
	porM1-as-2	5'-CGG ATC CTT GTC CAG ACC CGC ATG AGA T-3'

*porM1*	porM1-fw-hind	5'-ACA AGC TTG CTC TCA GCC GGT TTT CA-3'
	porM1-bw-hpa	5'-CAG TTA ACG AAC TGG GCG TTC ATG TGC-3'

*porM1*	porM1-51-sybr-fw	5'-GCT GTT TAC GAG CAC GGG C-3'
	porM1-51-sybr-bw	5'-TTG CGG TCC AGG GGG AAC-3'

*mspA*	mspATaqFW	5'-CGT GCA GCA GTG GGA CAC CTT-3'
	mspATaqBW	5'-CCA CGA TGT ACT TGG CGC GAC-3'
	mspATaqProbe	5'-FAM-TGG ACC GCA ACC GTC TTA CCC GTG AGT G-TAMRA-3'

*porM*	mfpqPCRfw	5'-CGT TCA GCA GTG GGA CAC CTT-3'
	mfpqPCRrev	5'-CCA CGG TGT ACT TGG CCC GGC-3'
	mfpqPCRprobe	5'-FAM-TGG ACC GCA ACC GGC TGA CCC GTG AGT G-TAMRA-3'

**Figure 2:**
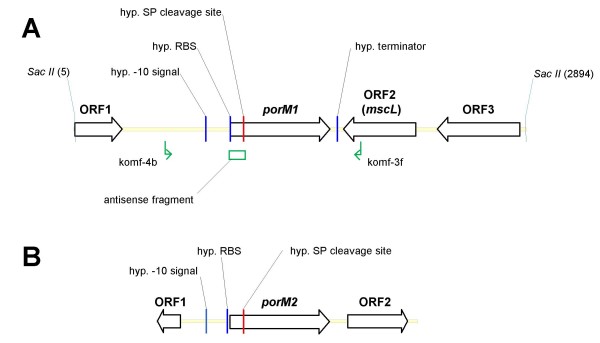
**Map of genomic regions containing *porM1 *from *M. fortuitum *10860/03 and *porM2 *from *M. fortuitum *10851/03**. Section A shows a 2895 bp region representing the insert of plasmid pSSp107. The insert includes the *porM1 *gene and three other ORFs. Up- and downstream to *porM1 *various nucleotide signal sequences were detected: -10 signal of a promoter (TATGTT), a ribosome binding site (RBS: GGAGA), a signal peptide recognition sequence (SP) of 81 bp and a hairpin structure, which could represent a terminator. Furthermore, the location of the antisense fragment selected for the generation of plasmid pSRr106 is indicated. Section B represents a 1697 bp region of *M. fortuitum *10851/03 containing *porM2 *and two other ORFs. Upstream to *porM2 *a -10 signal of a promoter (TACGTT), a ribosome binding site (AGGGAGAA) and a signal peptide recognition sequence (SP) of 93 bp were identified. Subsequences were predicted using the software packages MacVector™ 7.2.3 (Accelrys) and Lasergene (DNASTAR).

To clone porin genes from *M. fortuitum*, genomic DNA of *M. fortuitum *10860/03 was digested with *SacII*, and a 3000 bp fragment, which hybridised to the probe, was cloned in pIV2 and transformed into *E. coli*. Two clones (pSSp107 and pSSp108) containing porin sequences were isolated. Both plasmids were found to contain the same genomic region of 2895 bp, harbouring one porin gene. The inserts were sequenced by primer walking. Both strands of the porin genes and 400 bp of surrounding regions were sequenced at least twice. As shown in Figure 2A, the insert of the plasmids contained several open reading frames (ORFs), one of which was an ortholog of *mspA*. It contained 636 bp, encoding a protein of 211 amino acids with an N-terminal signal sequence of 27 amino acids, which was predicted using the SignalP 3.0 Server at http://www.cbs.dtu.dk/services/SignalP/[11]. The *in silico *analysis of the mature PorM1 (protein without signal peptide) showed a calculated molecular weight of the monomer of 19400 Da and an isoelectric point (pI) of 4.31.

A hypothetical -10 region of a promoter and a ribosome binding site (RBS) were identified upstream of the coding sequence. Downstream of the ORF a hairpin sequence was detected, which might function as a terminator (Figure 2A). It has to be noted that the sequence similarity between *M. fortuitum *and *M. smegmatis *was only restricted to the coding sequence. According to the designation of other mycobacterial porin genes and to the instructions of EMBL nucleotide sequence database, the gene was named *porM1 *(see Table 2 for accession no.).

**Table 2:** Nucleotide sequence similarity between *porM1 *and *porM2 *from members of the *M. fortuitum*-group and *mspA*.

Gene	Species	Nucleotide similarity index	Accession-no. to the EMBL nucleotide sequence database
*porM1*	*M. fortuitum *DSM 46621	88.2%	AJ880097
	*M. fortuitum *10851/03	88.4%	AJ880098
	*M. fortuitum *10860/03	87.4%	AJ874299
*porM2*	*M. fortuitum *10851/03	86.5%	AM295792
	*M. fortuitum *10860/03	86.5%	AM295793

Besides the porin gene, two other complete ORFs and part of another ORF were detected. ORF1 was interrupted by one of the *SacII *sites and showed a high similarity to a molybdopterin biosynthesis protein of *M. tuberculosis *CDC 1551 (accession no.: AAK 45260). ORF2 turned out to be a mechanosensitive channel orthologous to the gene *mscL *from *M. avium subsp. paratuberculosis *str. 10 (accession no.: NP 959854). ORF3 was similar to the hypothetical protein Rv0990c from *M. tuberculosis *H37Rv (accession no.: NP 215505). The entire cloned genomic region was blasted against the *M. tuberculosis *genome from the Sanger Institute database http://www.sanger.ac.uk/cgi-bin/blast/submitblast/m_tuberculosis to examine if the whole region is conserved between *M. fortuitum *and *M. tuberculosis*. However, only ORF1 and ORF2 possessed nucleotide identities higher than 60% showing that the region is not conserved among these mycobacteria.

**Figure 3:**
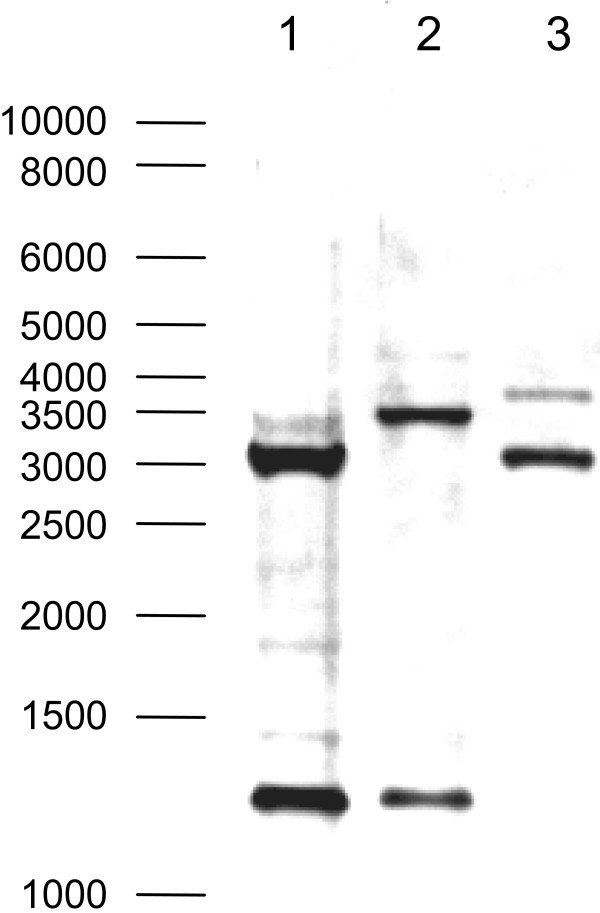
**Occurrence of porin genes in *M. fortuitum***. Chromosomal DNA of different strains was digested with *SacII *and analysed by Southern Blotting using a probe derived from the *porM1 *sequence. Lane 1: *M. fortuitum *10851/03; lane 2: *M. fortuitum *10860/03; lane 3: *M. fortuitum *DSM 46621.

A new probe derived from the *porM1 *sequence was used to detect porin genes in different *M. fortuitum *strains. The probe hybridised to two fragments of the *SacII*-digested genomic DNA of different *M. fortuitum *strains. However, the fragment size differed among different strains (Figure 3). Hence, the *M. fortuitum *genomes contain at least two porin genes.

Next, the presence of *porM1 *in other *M. fortuitum *strains was analysed. For this purpose, the *porM1*-specific primers komf-3f and komf-4b (Figure 2A and Table 1) were chosen to amplify a fragment of approximately 1250 bp, comprising the *porM1 *gene and its flanking regions. PCRs using a polymerase-mix with proofreading activity generated a fragment of the expected size in all strains. Several PCRs were performed and both strands of the different fragments were sequenced. *PorM1 *was detected in all three *M. fortuitum *strains, and the nucleotide sequences were submitted to the EMBL nucleotide sequence database (Table 2). The nucleic acid subsequences such as the -10 signal of a promoter, the RBS, the signal peptide of 81 bp and the hairpin structure were also present and were conserved among all strains tested (data not shown). As already indicated in Table 2, the nucleotide sequences of the gene *porM1 *differed among different strains of *M. fortuitum*.

**Figure 4:**
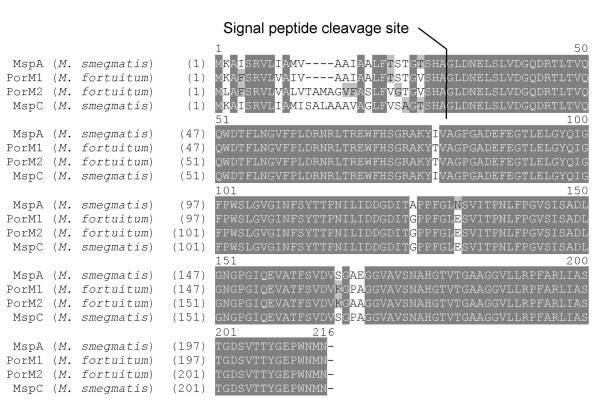
**Alignment of PorM1 and PorM2 from *M. fortuitum *and MspA and MspC from *M. smegmatis***. The start codon ATG and the stop codon TGA were chosen according to the sequence of *mspA*. The cleavage recognition site of the signal peptidase was predicted for PorM1, PorM2 and MspC using the SignalP 3.0 Server at http://www.cbs.dtu.dk/services/SignalP/[11]. The predicted signal peptide cleavage sites corresponded to the signal peptide cleavage site of MspA [6]. Identical amino acids are dark grey, similar amino acids are light grey and different amino acids are not shaded. For PorM1 and MspA an identity index of 94.8% was calculated, while PorM2 showed an amino acid identity of 90.7% to MspA.

The amino acid sequences of PorM1 among the *M. fortuitum *strains 10851/03 and 10860/03 including the type strain were identical (Figure 4). The mature PorM1 from *M. fortuitum *featured six amino acid substitutions compared to MspA.

Since the southern blot experiments had indicated the existence of two genes orthologous to *mspA *in *M. fortuitum*, we also attempted to clone and characterise the second porin gene. This porin gene, termed *porM2*, was amplified by PCR and cloned as a 918 bp fragment into the mycobacterial vectors pMV306 and pMV261, as described in the section Methods. The corresponding recombinant plasmids were named pSRa104 and pSRb103, respectively. Positive clones were confirmed by sequencing. As shown in Figure 2B, the insert of the plasmids contained an ORF of 648 bp, which turned out to be paralogous to the gene *porM1*. The 648 bp ORF encodes a protein of 215 amino acids with an N-terminal signal sequence of 31 amino acids, which was predicted using the SignalP 3.0 Server at http://www.cbs.dtu.dk/services/SignalP/[11]. The *in silico *analysis of the mature PorM2 showed a calculated molecular weight of the monomer of 19374 Da and a pI of 4.31, which were very similar to the calculated values of PorM1. A hypothetical -10 promoter sequence and a hypothetical RBS were located upstream of *porM2*. A hypothetical terminator sequence was, however, not detected (Figure 2B). The similarity between *porM1 *and *porM2 *from strains *M. fortuitum *10851/03 and 10860/03 on nucleotide level amounted to 94.1% and 95.3%, respectively. The *mspA *gene revealed to be more similar to *porM1 *(87.4% to 88.4% similarity) than to *porM2 *(86.5% similarity). Sequence comparison revealed that *porM2 *encodes a protein mainly differing from *porM1 *within the signal sequence. PorM2 from *M. fortuitum *10851/03 and 10860/03 exhibits an insertion of four amino acids and additional six amino acid exchanges within the signal peptide compared to PorM1 (Figure 4). Only one amino acid is replaced in the mature polypeptide [proline^165 ^(PorM1) with alanine^169 ^(PorM2)]. We sequenced a 1697 bp region comprising *porM2*, 500 bp of its upstream region as well as 549 bp downstream of *porM2*. The DNA sequences flanking the *porM1 *and *porM2 *genes revealed no similarity with exception of the 13 bp preceding the *porM*-coding sequences, which were identical in both regions. The 5' terminus of an ORF orthologous to a glycosyl transferase gene from *M. tuberculosis *CDC1551 (accession no.: AAK 48256) was detected upstream from *porM2*. An ORF orthologous to the gene for a pyridoxamine 5'-phosphate oxidase-related protein from *M. vanbaalenii *(accession no.: ZO 01208463) was present in the downstream region of *porM2 *(Figure 2B).

Using the primer pairs porM2-fw-hind and porM2-bw-hpa or porM2-rna-fw and porM2-rna-bw (Table 1), *porM2 *was also detected in other strains analysed. No product was obtained using the plasmid pSSp107 carrying *porM1 *as template, demonstrating the specificity of this PCR approach for *porM2*.

### *M. fortuitum *strains express less porin compared to *M. smegmatis*

**Figure 5:**
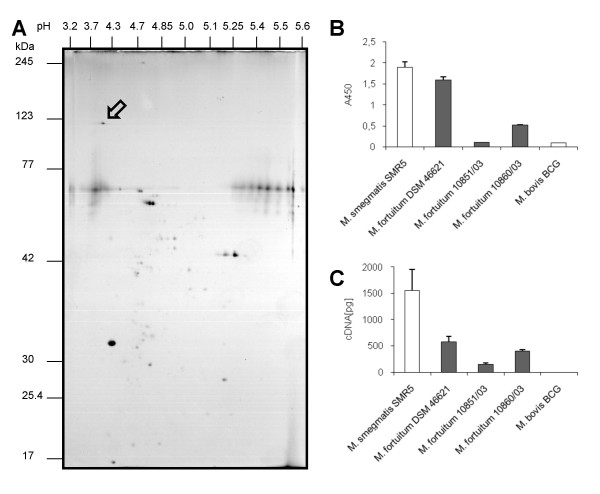
**Detection of PorMs in *M. fortuitum *and *M. smegmatis*. 2D-Electrophoresis, Western Blot, ELISA and qRT-PCR experiments proved PorMs to be expressed in the analysed strains**. Section A shows 2D-Electrophoresis of protein isolation from the strain *M. fortuitum *10860/03 using the detergent nOPOE. The arrow indicates the porin spot proven by Western Blot analysis (see Additional file 2). Section B and C show comparative analysis of porin expression among RGM. Expression of porin was detected by means of ELISA (B) and qRT-PCR (C). Each value represents the mean (± SD) of at least three independent experiments. B: Quantification of porin by means of ELISA in cell extracts of different mycobacteria using the polyclonal antibody pAk MspA#813. C: RT-Real-time-PCR quantification of porin mRNA in various RGM using specific primers and probes for *mspA *or *porM*, respectively.

The expression of the porins PorM1 and PorM2 were examined by 2D-Electrophoresis, Western Blotting, ELISA and qRT-PCR. For porin protein analysis, *M. fortuitum *pellets were lysed in POP05 (PBS 0.5% (w/v) n-octylpolyoxyethylene/0.2% EDTA) that was shown to selectively extract MspA [12]. For enhanced resolution and characterisation of the proteins, porin preparations were subjected to 2D-Electrophoresis. As shown in Figure 5A, about 50 protein spots were detected on the 2D-gel in *M. fortuitum *POP05 cell extracts. Western blot experiments with identical gels showed only one defined spot detected by the antiserum pAK MspA#813 [6] (see Additional file 2). The protein had an apparent molecular mass of approximately 120 kDa, the expected size of the oligomeric porin, and an apparent pI of about 4, which corresponded well to the predicted pI of the mature protein of 4.31. Oligomers of the porin were readily detected in cell extracts of all *M. fortuitum *strains as well as in extracts from *M. smegmatis *that served as a positive control. After extended exposition times, the monomer of the porin was also detected on Western Blots (data not shown). The Western Blots showed considerable differences in porin protein expression among the analysed strains (see Additional file 3). Additionally, ELISA experiments with POP05 extracts were performed to quantify the amount of porin in different strains. Different dilutions of cell extracts from the various strains were loaded into the wells of a microtiter plate and porins were detected using the polyclonal antibody pAK MspA#813. Since *M. bovis *BCG does not possess orthologous porins [6], extracts of *M. bovis *BCG were employed as a control to detect the background. Amounts higher than 5 μg per well turned out to be inapplicable due to saturation effects, and the detection of porin in cell extracts failed at concentrations of about 0.04 μg per well. Therefore, the most eligible working range turned out to be 1 μg of cell extract per well. Indeed, the amount of porin differed in various strains. The highest amount of porin was detected in the internal control *M. smegmatis *SMR5. The type strain *M. fortuitum *DSM 46621 exhibited porin amounts close to those of *M. smegmatis*, whereas the other two strains showed clearly decreased porin amounts (Figure 5B). Notably, *M. fortuitum *10851/03 exhibited the lowest amount of porin very close to the background, which was represented by the control *M. bovis *BCG.

Comparative expression analysis was also performed by means of quantitative reverse transcription polymerase chain reaction (qRT-PCR) using sequence-specific primers and probes (Table 1). The values were compared to porin expression in *M. smegmatis*. Because of the high degree of sequence conservation of the two paralogs *porM1 *and *porM2*, a qRT-PCR approach was established using primers and a dually labelled probe that hybridised to a region where both genes are identical (*porM1 *amplicon: nucleotide 132–232 and *porM2 *amplicon nucleotide 144–244, see also Table 1). This PCR approach enabled the quantification of the overall expression of the paralogs. As was already proven by the ELISA results, the highest porin mRNA expression was measured in *M. smegmatis*. It showed transcription rates about twice as high as the highest level among the *M. fortuitum *strains, which was detected in the type strain *M. fortuitum *DSM 46621. *M. fortuitum *10851/03 exhibited the lowest transcription rate among all *M. fortuitum *strains (Figure 5C). The quantification of *porM *mRNA as well as the protein isolated from the various strains demonstrated consistence of transcriptional and translational levels and underlined the differential porin expression among the analysed *M. fortuitum *strains.

MspA was shown to be accessible on the cell surface of *M. smegmatis *by using pAK MspA#813 [8]. Since the expression analysis showed a differing amount of porin in *M. fortuitum *strains, *M. fortuitum *DSM 46621 and *M. fortuitum *10860/03 (the strains with the highest porin expressions) were employed for detection of porins at the surface of intact mycobacteria. Porins were accessible at the surface of intact cells of *M. fortuitum *and were detected by the porin-specific antibody. Significantly higher absorption at 450 nm (P < 0.001) was measured for *M. fortuitum *DSM 46621 as well as *M. fortuitum *10860/03 when compared to the relative backgrounds (see Additional file 4).

### *PorM *expression in the porin-deficient mutant strain *M. smegmatis *ML10 leads to improved growth

Heterologous expression of *porM1 *as well as *porM2 *was performed in the porin mutant strain *M. smegmatis *ML10 (Δ*mspA*; Δ*mspC*) to prove the functionality of encoded porins. For this purpose, the plasmids pSRa102 and pSRa104 harbouring *porM1 *and *porM2*, respectively, were introduced to *M. smegmatis *ML10. The plasmid pSSa100 [13] containing *mspA *was employed as a positive control.

**Figure 6:**
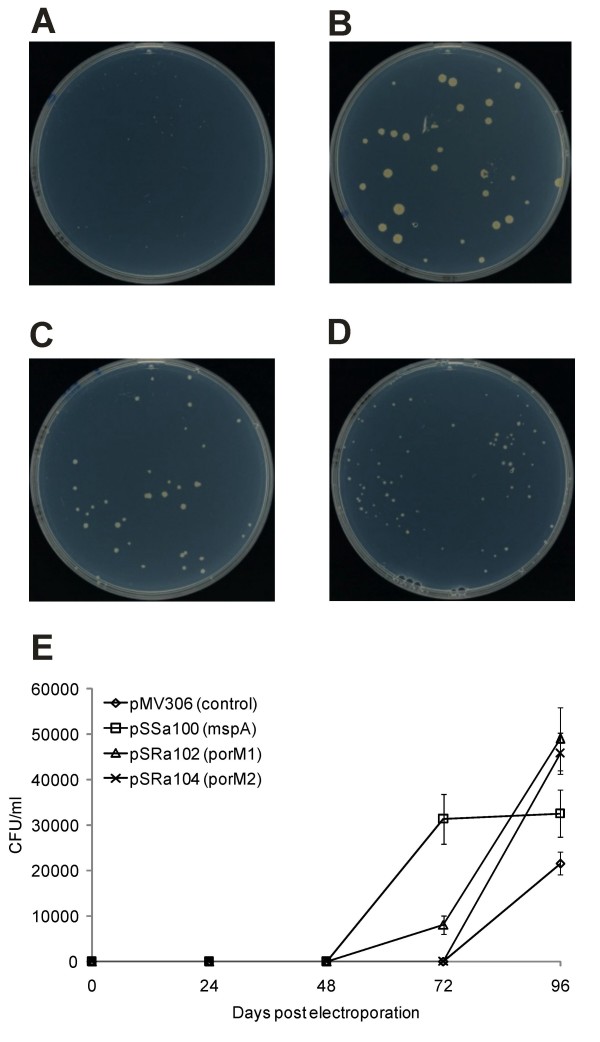
**Complementation of the porin-deficient mutant strain *M. smegmatis *ML10 with *porM1 *and *porM2***. *M. smegmatis *ML10 was transformed with the control vector pMV306 (A), the *mspA*-containing plasmid pSSa100 (B), the *porM1*-containing plasmid pSRa102 (C) and the *porM2*-containing plasmid pSRa104 (D). After electroporation of the plasmids, dilutions of the transformed bacteria were plated onto Mycobacteria 7H11 agar with 25 μg ml^-1 ^kanamycin and incubated for four days. Panel (E) illustrates the result of an independent experiment showing the time course of the appearance of the colonies on Mycobacteria 7H11 agar with 25 μg ml^-1 ^kanamycin during four days after plating of a 1:10 dilution of the electroporated cells.

First, the growth on Mycobacteria agar plates was quantified during four days after electroporation. The growth was compared to a strain harbouring only the vector pMV306. As it is shown in Figure 6A to 6D, heterologous expression of *mspA*, *porM1 *and *porM2 *led to enhanced growth of complemented strains compared to the control. Figure 6A and 6B illustrate the growth retardation of strain *M. smegmatis *ML10 (pMV306) compared to the *mspA*-complemented strain *M. smegmatis *ML10 (pSSa100). The growth of *M. smegmatis *ML10 was ameliorated by both, plasmid pSRa102 as well as plasmid pSRa104 (Figure 6C and 6D), although the complementation of the growth defect by these plasmids was less pronounced than by *mspA *expression using pSSa100. Heterologous expression of *porM1 *in the *M. smegmatis *mutant (Figure 6C) resulted in better growth than heterologous expression of *porM2 *(Figure 6D).

The quantification of growth rates of the strains by cfu-counting confirmed these conclusions (Figure 6E). The strain complemented with *mspA *(containing pSSa100) had reached its final number of colonies after 72 hours. The transformant complemented with *porM1 *(containing pSRa102) also showed visible colonies after 72 hours, it had, however, not yet reached its final number of colonies after this time period. The strains containing pSRa104 (carrying *porM2*) and the empty vector pMV306 both showed visible colonies not until 96 hours, but ML10 (pSRa104) outnumbered ML10 (pMV306).

### Knock-down of *porM *expression by RNA antisense technique as well as over-expression of *porM1 *or *porM2 *affect the growth rate of *M. fortuitum*

Knock-down of porin expression in *M. fortuitum *was performed by generating the plasmid pSRr106, which carries a *porM *antisense fragment (see Figure 2A) under the control of the *hsp60 *promoter. The employed antisense sequence was first tested for non-specific binding performing a blast search at http://blast.ncbi.nlm.nih.gov/Blast.cgi. The analysis ensured that the antisense fragment specifically binds to *mspA *class porins, such as *porMs *and did not show any hits to other sequences deposited in the database.

The efficiency of down-regulation via RNA antisense technique was proven by means of SYBR Green qRT-PCR using strain 10860/03. As shown in Additional file 5, the knock-down strain carrying the plasmid pSRr106 showed about four times lower porin expression compared to the control strain harbouring the vector pSHKLx1.

In order to over-express *porM *genes in *M. fortuitum*, the coding sequences of *porM1 *from strain *M. fortuitum *10851/03 and of *porM2 *from strain 10860/03 were inserted downstream of the *hsp60 *promoter in the vector pMV261 to generate plasmids pSRb101 and pSRb103, respectively.

**Figure 7:**
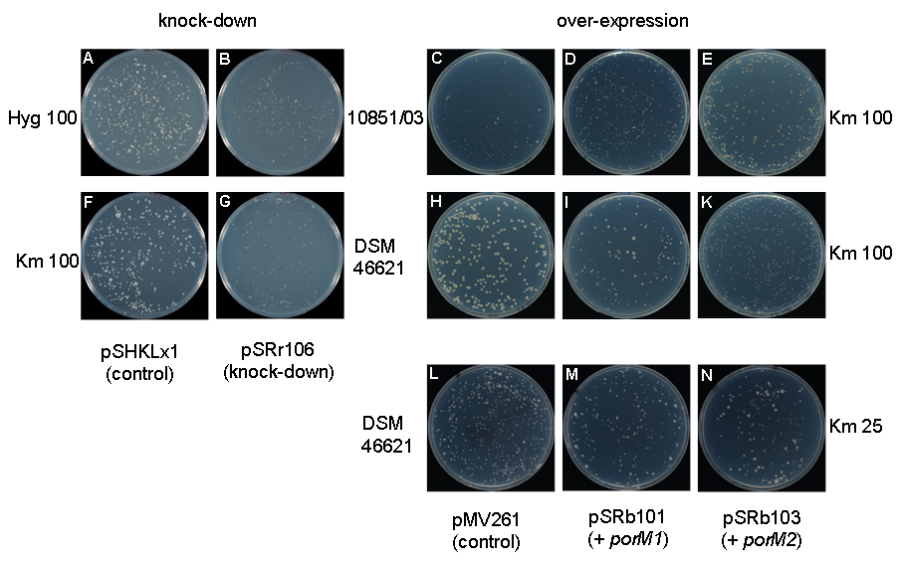
**Effect of down-regulation and over-expression of *porM1 *and *porM2 *on the growth of *M. fortuitum***. *M. fortuitum *strains 10851/03 (A-E) and DSM 46621 (F-N) were electoprorated with plasmids pSHKLx1 (A, F), pSRr106 (B, G), pMV261 (C, H, L), pSRb101 (D, I, M) or pSRb103 (E, K, N), plated on either 100 μg ml^-1 ^hygromycin (A, B), 100 μg ml^-1 ^kanamycin (C-K) or 25 μg ml^-1 ^kanamycin (L-N) and incubated for four days.

We first studied the impact of the modified *porM *expression rates on the growth of bacteria freshly transformed with plasmids pSRr106, pSRb101 and pSRb103 as well as with the empty vectors pSHKLx1 and pMV261, serving as negative controls. Strains transformed with pSHKLx1 or pSRr106 were either selected by adding kanamycin (100 μg ml^-1^) or hygromycin (100 μg ml^-1^) to the agar, while transformants electroporated with pMV261, pSRb101 or pSRb103 were selected by addition of kanamycin (100 μg ml^-1^). The clearest results were obtained with strains 10851/03 and DSM 46621 and are displayed in Figure 7(A, B, C–E, F, G, H–K). Knock-down of *porM *expression in both strains resulted in considerable growth reduction (Figure 7A, B and 7F, G) substantiating an important role of porins for the growth of *M. fortuitum*. This was further supported by the growth pattern of the 10851/03 derivatives over-expressing *porM1 *or *porM2 *(Figure 7C–E). Compared to 10851/03 containing the empty plasmid pMV261, both derivatives over-expressing *porM *genes brought about a slight increase in average colony size on plates containing 100 μg ml^-1 ^kanamycin. This effect was more pronounced in 10851/03 over-expressing *porM2 *than in the strain over-expressing *porM1*. In DSM 46621 the porin over-expression had an adverse effect on growth upon plating on 100 μg ml^-1 ^kanamycin (Figure 7H–K). In order to figure out if this growth decrease was caused by an increased antibiotic uptake, we then plated the over-expressing DSM 46621 derivatives and the control on plates containing only 25 μg ml^-1 ^kanamycin (Figure 7L–N). Under these conditions, the over-expression of *porM *genes slightly enhanced the growth. Again the increase in average colony size was more pronounced upon over-expression of *porM2*.

## Discussion

In recent studies, our research has concentrated on the impact of the cell wall permeability on growth and intracellular persistence of mycobacteria. We were able to show that the porin pathway affects the intracellular persistence of different species in different ways. The findings suggest that intracellular persistence of mycobacteria depends, inter alia, on the balance between "walling-off" towards the hostile environment and the uptake of required compounds in the nutrient-depleted phagosomal environment [5,13,14]. To further examine this hypothesis, we are searching for more appropriate models. Different views have been expressed among scientists about whether *M. smegmatis *could serve as an appropriate model to study aspects related to virulence of highly pathogenic mycobacteria. A notable number of *M. tuberculosis *genes that are related to virulence but also play a housekeeping role share closely related orthologs in *M. smegmatis*. In the case of common mycobacterial genes, *M. smegmatis *was suggested as an appropriate model organism [15,16]. On the other hand, the physiological differences between *M. smegmatis *and *M. tuberculosis *were mentioned to narrow down the significance of direct comparisons [17]. Mutagenesis of porin genes in *M. smegmatis *allows the investigation of the impact of cell wall permeability on persistence. However, more appropriate models for such studies must naturally be able to survive and multiply intracellularly. Additionally, they must possess a known class of porin. These conditions are fulfilled by *M. fortuitum*, which was recently suggested as a model *Mycobacterium *[9]. This species is able to infect and grow in phagocytic cells [2,3] and also possesses porins orthologous to MspA. We therefore decided to identify and characterise porin genes from *M. fortuitum*.

The results of this study show that different strains – including the type strain – of *M. fortuitum *possess orthologous porins of the MspA class. The amino acid sequences of PorM1 and PorM2 are highly conserved among the strains, whereas there is variability in their nucleotide sequence. PorM1 and PorM2 have the same apparent molecular mass as MspA and MspC, respectively. They are accessible at the surface of *M. fortuitum*. In detergent extracts of *M. fortuitum *mature oligomers of PorMs were detected, similar to *M. smegmatis *porin oligomers. As oligomer formation is necessary for channel activity [18], it can be concluded that *M. fortuitum *porins form functional pores in the OM. Mature PorM1 from *M. fortuitum *differs at only six amino acid positions from MspA. According to the studies of Faller *et al. *[7] and Mahfoud *et al. *[19] about the structure of MspA and its topology in the OM of *M. smegmatis*, it can be assumed that the amino acid replacements between PorM1 and MspA do not significantly affect the general porin structure. Remarkably, most of the exchanges are restricted to those residues, which are also variable within the Msp family. For example, the replacement of alanine^138 ^with proline in the extracellular loop L9 between the β-sheets 9 and 10 supports the tight turn between the β-sheets and the change of direction. Interestingly, PorM2 does not feature the mentioned exchange of alanine with proline, which is the only amino acid exchange in the mature protein between PorM1 and PorM2. Faller *et al. *[7] proposed that the adjacent replacement of serine^163 ^with lysine changes the antigenic properties of MspD compared to the other isomers. Although PorMs have the same exchange, they were readily detectable using a polyclonal antibody raised against MspA. The exchange of asparagine^129 ^with glutamic acid within the periplasmatic loop L6 introduces a negative charge into the channel and may thus change the permeation properties slightly [7]. The replacement of isoleucine^76 ^with threonine within the β-sheet β3 should not affect the protein structure either, since both amino acids are C-beta branched amino acids and it is more favourable for them to lie between β-sheets [20].

The capacity of the encoded porins PorM1 and PorM2 to fulfil the function of a porin was tested in complementation experiments by introducing these genes into the double mutant strain *M. smegmatis *ML10 (Δ*mspA; *Δ*mspC*) and observing the growth rate. Interestingly, *porM1 *had a stronger complementation effect than *porM2*, which was indicated by faster appearance of colonies and larger colony sizes on plates after electroporation. This may be explained by the higher similarity of *porM1 *to *mspA*, which represents the main porin gene in *M. smegmatis *[8]. The antiserum raised against MspA binds well to PorMs, and the growth defect of the mutant strain *M. smegmatis *ML10 is reduced after complementation with *porM1 *and *porM2*. All mentioned features indicate similar functions and characteristics of the porins from *M. smegmatis *and *M. fortuitum*.

As mentioned above, mature PorM2 only differs from the mature PorM1 in one amino acid. More remarkable differences occur in the signal peptide of the two porins. The calculated cleavage site (SHA-GL) of the signal peptides of PorM1, PorM2, MspA and MspC is identical. However, the length of the signal peptides differs. While PorM1 and MspA have signal peptides composed of 27 amino acids, PorM2 and MspC possess extended signal peptides consisting of 31 amino acids. The length and primary structure of the signal peptide could be important for the transport and integration of the particular porin to the mycobacterial OM. It will be very informative to analyse the different regulation mechanisms determining the importance of the two genes for growth under different environmental conditions.

It was interesting that the expression of *porM *genes both at the transcriptional level and at the translational level consistently differed among the analysed strains as shown by the three employed approaches (Western Blot, ELISA and qRT-PCR). The results of both quantitative assays show the lowest porin expression among *M. fortuitum *strains in 10851/03 followed by 10860/03 and the type strain. The use of a polyclonal antibody, which recognises different epitopes of the protein and the consistency among the results of three different approaches allows drawing the conclusion that the porin expression in *M. fortuitum *is lower compared to *M. smegmatis *and also varies between the different strains. The high sequence conservation of the two paralogs PorM1 and PorM2 does not allow their expressions to be distinguished. Therefore, we consider the expression rates as overall values of both paralogs. As shown by qRT-PCR and ELISA, the porin expression in different strains of *M. fortuitum *was significantly lower than that of *M. smegmatis*. It was shown that *M. smegmatis *possesses 1000 MspA-like pores per μm^2 ^cell wall [21]. Since the analysed strains of *M. fortuitum *exhibited a clearly lower *porM *expression both at the transcriptional and the translational level, the amount of pores in the cell wall of *M. fortuitum *must be distinctly lower than 1000 pores per μm^2 ^cell wall. According to our results, the amount of MspA-like pores in the analysed strains of *M. fortuitum *varies between 600 in *M. fortuitum *DSM 46621 and less than 100 per μm^2 ^cell wall in *M. fortuitum *10851/03, which exhibits the lowest amount of porin at all. It is interesting that the strain exhibiting the lowest porin expression is identical with the strain showing the slowest growth rate. This finding supports the hypothesis that porins play an important part in determining the generation time of mycobacteria.

To investigate the impact of the porins PorM1 and PorM2 on the growth rate of *M. fortuitum*, we generated strains over-expressing *porM1 *or *porM2*. Additionally, *M. fortuitum *knock-down strains were generated by antisense technique. This technique has contributed to the clarification of the function of many mycobacterial genes. Advantages are the possibility to analyse essential genes whose mutagenesis would be lethal and to repress genes present in several copies. Some examples of the application of the antisense technique in mycobacteria are the repression of *ahpC *from *M. bovis *[22], *dnaA *from *M. smegmatis *[23], FAP-P from *M. avium *subsp. *paratuberculosis *[24], *pknF *from *M. tuberculosis *[25] or MDP1 from *M. bovis *BCG [26]. A further advantage of knocking-down genes by antisense technique can be the possibility to repress paralogous genes in the same bacterium. As described in Dryselius et al. [27], the most effective region for antisense inhibition is the region covering the Shine-Dalgarno Sequence and the start codon. The authors furthermore show, that antisense RNAs of only 10–12 bases exhibit strong inhibitory effects on gene expression. The antisense fragment used in this study is identical to the corresponding region of *porM1*. While it displays a homology of 71.4% to *porM2*, the antisense fragment and *porM2 *still exhibit long stretches of identical nucleic acid sequences. Of particular importance is the similarity in the beginning of the antisense fragment covering the Shine-Dalgarno Sequence and the start codon (40 bp, 95% identity). We therefore are convinced that a down-regulation of both, *porM1 *as well as *porM2*, may be achieved using the strategy described in this study. Deletion- or insertion mutagenesis of either *porM1 *or *porM2 *might result in complementation of the deleted porin gene by the remaining one. Such an effect has been observed in *M. smegmatis*, where the deletion of the *mspA *gene caused the activation of the transcription of *mspB *and/or *mspD *[28]. Mutagenesis of both porin genes in the same derivative, on the other hand, would probably restrain the diffusion across the OM to an extent compromising cellular functions.

The effects of an over-expression of porin in our *M. fortuitum *strains depended on characteristics of the strains as well as the amount of kanamycin added to the medium. The over-expression of *porM1 *and *porM2 *showed the most considerable influence on growth rate in strain 10851/03. Among the tested strains, 10851/03 has the slowest growth rate and produces least porin. Therefore, this strain probably benefits most from a better nutrient supply caused by porin over-production. Otherwise, the adverse effect of kanamycin on the growth rate was most pronounced in strain DSM 46621, which expresses the highest amount of porin among the analysed strains. Disposing of a relatively high amount of porin, this strain probably takes less advantage of an ameliorated nutrient supply and instead suffers most from more kanamycin diffusion into the cells. When the kanamycin concentration in the plates was reduced to 25 μg ml^-1^, the over-expressing DSM 46621 derivatives did not show any growth inhibition compared to the control strain and even had a slight growth advantage. It seems that at this kanamycin concentration the beneficial effects of better nutrient influx slightly exceed the adverse effects of better antibiotic influx. The changes in growth behaviour in 10851/03 as well as in DSM 46621 were more pronounced upon over-expression of *porM2 *compared to over-expression of *porM1*. The down-regulation of the expression of PorM1 together with PorM2 by antisense-technology reduced the growth of both *M. fortuitum *strains to a similar and very low level suggesting that lack of porins in the knock-down strains strongly impairs the nutrient supply.

Our observations point to a passage of kanamycin through the PorM porins. Studies performed with *M. smegmatis *gave rise to contrarious conclusions [29,30]. Stephan et al. [29] observed no reduction of kanamycin resistance in a *mspA *mutant compared to the *M. smegmatis *wild type strain and Danilchanka and colleagues [30] had postulated using structural models that due to its size kanamycin cannot diffuse through the MspA porin. These differing results may in part be explained by the use of different experimental systems. Stephan et al. [29] employed a mutant strain, while we observed differential effects of kanamycin only in over-expressing strains. Furthermore, Stephan et al. [29] performed their studies with *M. smegmatis *and we observed strong strain-dependent variations even among different isolates within the same species. The amino acid exchanges occurring between MspA on the one hand and PorM1 and PorM2 on the other hand may be responsible for differences in channel properties of these porins and influence their permeability for kanamycin.

As we discussed earlier, the growth rate of mycobacteria may contribute to their pathogenicity [14]. Hence, it can be suggested that the low porin expression in *M. fortuitum *strains isolated from human patients compared to saprophytic species of RGM like *M. smegmatis *contributes to higher pathogenicity caused by an enhanced ability to multiply intracellularly. Interestingly, it was shown that the *mspA *expression in *M. smegmatis *is specifically downregulated at acidic pH [31]. Moreover, the *M. tuberculosis *porin OmpATB, which belongs to the OmpA class of porins has been shown to be necessary for the persistence in the acidic milieu enabling *M. tuberculosis *to respond to reduced environmental pH [32,33]. Although the MspA like porins do not belong to the OmpA class of porins, the results of these studies underline the role of porins concerning the intracellular persistence of mycobacteria.

An interesting result from various genome-sequencing projects of mycobacteria is that genome sizes of RGM and the pathogenic slow-growing mycobacteria largely differ. Highly pathogenic species like *M. tuberculosis *and *M. leprae *have genome sizes of about 4.4 Mb and 3.27 Mb, respectively. On the other hand, *M. smegmatis *has a genome size of about 7 Mb, which is similar to that of the related actinomycete *Streptomyces coelicolor*. Brosch *et al. *[34] reviewed different data such as 16S rRNA sequences or genome sizes and suggested that the branch of slow-growing mycobacteria represents the part of the genus that has evolved most recently. They proposed that the loss of genes rather than gain of genetic material by horizontal transfer contributed both to the pathogenicity of slow-growing mycobacteria and to the fine-tuning of their virulence. Loss of efficient porins of the MspA class or a decreased density of porins in the OM plays an important role to "wall-off" toward the hostile phagosomal environment and thus is of particular importance for the evolution of a successful intracellular pathogen. The presence of several copies of porin genes and, in turn, a high density of efficient porins in the OM of *M. smegmatis *would provide a selective advantage for saprophytes. A decreased amount of efficient porins in the OM of pathogenic RGM like *M. fortuitum *may represent an evolutionary intermediate stage between saprophytic mycobacteria like *M. smegmatis *and the highly pathogenic slow-growing mycobacteria.

## Conclusion

Our study provides detailed information about porin genes of the *mspA *class in *M. fortuitum *and their importance for the growth rate and susceptibility to antibiotics. Our future studies will concentrate on the elucidation of the role of PorM1 and PorM2 in survival and replication of phagocytosed *M. fortuitum*.

## Methods

### Bacterial strains, cell lines and plasmids

Mycobacterial strains (Table 3) were grown in Middlebrook 7H9 medium (BD Biosciences, Heidelberg, Germany), supplemented with 0.05% Tween 80 and either ADC (BD Biosciences) or DC (2 g glucose, 0.85 g NaCl, in 100 ml H_2_O) at 37°C without shaking, or on Mycobacteria 7H11 agar (BD Biosciences), supplemented with ADC (BD Biosciences). For selection of recombinant mycobacteria, media were supplemented when required with 25 to 100 μg ml^-1 ^kanamycin or 100 μg ml^-1 ^hygromycin B. *E. coli *DH5α was grown in LB medium at 37°C [35]. Media were supplemented with 100 μg ml^-1 ^kanamycin or 200 μg ml^-1 ^hygromycin B for selection of recombinant *E. coli*. All plasmids used in this study are described in Table 4.

**Table 3:** Mycobacterial strains used in this work.

Strains	Characteristics	Reference
*M. smegmatis *SMR5	*M. smegmatis *mc^2^155 derivative, SM^R^	[42]
*M. smegmatis *ML10	SMR5 derivative, Δ*mspA *and Δ*mspC*	[4]
*M. fortuitum *DSM 46621	Type strain; HYG^R^	
*M. fortuitum *10851/03	Human patient isolate	This study
*M. fortuitum *10860/03	Human patient isolate; HYG^R^	This study
*M. bovis *BCG Copenhagen	Vaccine strain	

HYG: hygromycin; SM: streptomycin

### Measurement of growth rates in broth culture

To compare the growth rates of *M. fortuitum *strains, Middlebrook 7H9/DC medium was inoculated with preparatory cultures to obtain an initial OD_600 _of 0.02. During 16 days, the optical densities of the cultures were measured daily. Growth of the strains was monitored by quantification of the ATP content of the cultures with the luminescence-based kit BacTiter-Glo™ Microbial Cell Viability Assay (Promega). The luminescence was reported as relative light units (RLU) with the microplate luminometer LB96V (EG&G Berthold) [36].

### Molecular biology techniques and *in silico *analysis

Common molecular biology techniques were carried out according to standard protocols [35] or according to the recommendations of the manufacturers of kits and enzymes. Transformation of *E. coli *was performed according to the method of Hanahan [37]. PCR reactions were performed with the following kits: Taq DNA Polymerase (MBI Fermentas, St. Leon-Roth, Germany), BC Advantage GC Polymerase Mix (Takara Bio Europe S.A., Gennevilliers, France), BIO-X-ACT Short Mix and BIOTAQ DNA Polymerase (Bioline GmbH, Luckenwalde, Germany). Sequencing reactions were performed by using the Prism Big Dye™ FS Terminator Cycle Sequencing Ready Reaction Kit from PE Applied Biosystems (Darmstadt, Germany).

Protein and nucleotide sequence analysis such as identification of DNA subsequences (e.g. promoters and terminators) was performed using the software packages MacVector™ 7.2.3 (Accelrys, Cambridge, UK) and Lasergene (DNASTAR, Inc., Madison, WI, USA). Signal peptides were predicted using the SignalP 3.0 Server at http://www.cbs.dtu.dk/services/SignalP/[11].

Phylogenetic relationships among the RGM were analysed using the program ClustalW in the MacVector™ 7.2.3 package. Before analysing the phylogenetic relationships, sequences were trimmed in order to start and finish at the same nucleotide position for all employed strains. Phylograms were obtained from nucleotide sequences using the neighbour-joining method with Kimura 2-Parameter distance correction [38].

### Cloning of *porM1 *and *porM2 *from *M. fortuitum *and their detection in other strains of *M. fortuitum*

In order to clone porin genes, genomic DNA from *M. fortuitum *was digested to completion with the restriction enzyme *SacII *and separated by agarose gel electrophoresis. The DNA was then transferred to the Hybond+ membrane (GE Healthcare, Munich, Germany) as described by Sambrook and Russell [35]. Porin genes were detected by means of Fluorescein-labelled probes using the primer pairs hpor and npor or mf-4IV-fw and mf-4-bw (Table 1) and the PCR Fluorescein Labelling Kit (Roche, Mannheim, Germany) according to the manufacturer's instructions. The region around 3000 bp that was shown to hybridise to the probe was isolated out of the gel and was ligated into the unique *SacII *site of the plasmid pIV2 [39]. After transformation of *E. coli *DH5α, clones were screened by Dot Blot analysis. Inserts of two positive recombinant plasmids, pSSp107 and pSSp108, were sequenced. The inserts contained *mspA*-related sequences referred to as *porM1*. Identification of orthologous genes among other members of *M. fortuitum *was performed by PCR using the primers komf-3f and komf-4b (Table 1), which were derived from the cloned genomic region of *porM1*.

For the cloning of *porM2*, genomic DNA from *M. fortuitum *10851/03 DNA was digested with the restriction enzyme *SmaI *and a 4200 bp *SmaI *fragment that had shown to hybridise to the Fluorescein-labelled probe before was eluted from the agarose gel and ligated into the *SmaI *site of pLITMUS38 (New England Biolabs, Frankfurt, Germany) and clones were screened as mentioned above. The insert of the only positive clone was sequenced. A 181 bp sequence similar to the 3' terminus of the coding sequence of *porM1 *was identified, while the following 256 bp of the 3' flanking region showed no similarity to the *porM1 *flank. A PCR primer within the *porM2 *flanking region (porM2-51-bw) and another primer hybridising to the first 19 bp of the *porM1 *coding sequence (porM2-51-fw) were used to amplify *porM2 *sequences (Table 1). After sequencing the PCR product, a reverse PCR approach was adapted to discover the 5' flanking region of *porM2 *including its start. Therefore, genomic DNA of *M. fortuitum *10851/03 was digested with *NcoI*. The DNA fragments were circularised by ligation. Then a PCR was performed using the reverse primers porM2-rev-1 and porM2-rev-2 (Table 1) and the product was sequenced to obtain a complete sequence of *porM2 *and its flanking regions. The primers porM2-fw-hind (located 268 bp upstream of the *porM2 *coding sequence [CDS]) and porM2-bw-hpa (located directly downstream of the *porM2 *cds) (Table 1) were derived from the sequence mentioned and were chosen to amplify and clone *porM2 *and its regulatory sequences. The 918 bp product was cloned into the *HindIII*/*HpaI *restriction sites of the integrative mycobacterial vector pMV306 [40] and the shuttle vector pMV261 [40] to generate the recombinant plasmids pSRa104 and pSRb103, respectively. Positive clones were verified by sequencing. *PorM2 *was detected in other strains using the primer pairs porM2-fw-hind and porM2-bw-hpa or porM2-rna-fw and porM2-rna-bw (Table 1).

### Detection of porins by Western Blot and 2-D Electrophoresis

*M. smegmatis *MspA as well as porins from *M. fortuitum *were extracted in PBS buffer supplemented with 0.5% (w/v) n-octylpolyoxyethylene (nOPOE, Bachem, Heidelberg) and 0.2% EDTA (POP05), slightly modifying the method of Heinz and Niederweis [12]. Mycobacteria were grown to an OD_600 _of up to 1. Subsequently, about 150 mg of mycobacteria (wet weight) were washed twice in PBS buffer supplemented with 0.2% EDTA. Pellets were resuspended in POP05 using a ratio of 200 μl POP05 per 100 mg mycobacteria and were incubated at 100°C for 30 min. Afterwards, cell debris was sedimented by centrifugation at 27,000 × g and 4°C and the supernatant was transferred to a new tube. Quantification of protein samples was carried out using the BCA Protein Assay Reagent Kit (Pierce, Rockford, IL, USA). Western Blot analysis was performed using the antiserum pAK MspA#813 as described previously [13]. For 2D-analysis, about 75 μg of protein was precipitated by acetone and pellets were washed with 70% acetone to desalt the sample. Afterwards pellets were resuspended in 200 μl Rehydration solution (8 M Urea, 0.5% CHAPS, 0.2% DTT, 0.5% Pharmalyte, 0.002% Bromphenol blue), incubated for 5 h at room temperature and loaded on IPG strips pH 3–5.6 NL, 11 cm (GE Healthcare). The strips were focused on an Ettan pIGphorII unit and the second dimension was run on vertical 10% SDS-PAGE gels using the Ettan Daltsix electrophoresis unit (GE Healthcare) according to the manufacturer's instructions. The gels were silver-stained using Roti-Black P (Carl Roth GmbH, Karlsruhe, Germany). The porin was detected by Western Blotting as mentioned above.

### Differential expression analysis of porins by qRT-PCR and ELISA

Expression of porin genes in the different strains was determined by means of qRT-PCR using the Mx3000P™ Real-time PCR System (Stratagene, La Jolla, CA, USA) or the StepOnePlus™ Real-Time PCR-System (Applied Biosystems). Mycobacteria were grown to an OD_600 _of 0.8 and RNA was extracted according to the method of Bashyam and Tyagi [41]. 1 or 5 μg of the RNA was treated prior to qRT-PCR with RNase-Free DNase (Fermentas GmbH, St. Leon-Roth, Germany). Reverse transcription of mycobacterial RNA was carried out using the RevertAid™ M-MuLV Reverse Transcriptase (Fermentas GmbH) and hexamers or the Access RT-PCR System (Promega, Mannheim, Germany) according to the manufacturer's protocols. The porin cDNA from *M. smegmatis *SMR5 [42] and *M. fortuitum *was quantified either by amplifying a fragment of about 100 bp using the primers (mspATaqfw, mspATaqbw, mfpqPCRfw and mfpqPCRrev) as well as TaqMan-probes (mspATaqProbe and mfpqPCRprobe) or the primers porM1-51-sybr-fw and bw based on SYBR Green detection chemistry (Table 1). The qPCR reactions were performed using the SensiMix DNA Kit (Quantace Ltd., Berlin, Germany) or the Access RT-PCR System (Promega) according to the manufacturer's protocol. TaqMan quantification was carried out by running a first step at 95°C for 10 min followed by 40 cycles with 30 s at 95°C and 1 min at 58°C. SYBR Green quantification was performed by initial 10 min at 95°C followed by 40 cycles with 15 s at 95°C, 10 s at 58°C and 20 s at 72°C. Afterwards, the amplicon's melting temperature was determined ramping the temperature from 60°C to 90°C by 0.5°C steps and acquiring the fluorescence signal. cDNA amounts were determined by three measurements for each sample using a calibration curve established with known amounts of linearised pSSa100 [13] in case of *M. smegmatis *or pSSp107 in case of *M. fortuitum*. DNase treated and non-reverse-transcribed controls were performed with the same samples to guarantee the absence of contaminating genomic DNA.

In addition to the qRT-PCR experiments, the amount of porin in isolates of *M. fortuitum *and *M. smegmatis *was determined by Enzyme-Linked Immunosorbent Assay (ELISA). Protein was isolated from mycobacteria using the detergent nOPOE as described above. The isolated protein (15 μl corresponding approximately to 25 μg) was diluted in 50 mM NaHCO_3_, pH 9.6 to yield a protein concentration of 1 μg/100 μl. Aliquots (100 μl) of the sample and dilutions thereof were loaded to wells of a Nunc-Immuno Maxisorp Module (Nalgene Nunc International, NY, USA). After incubating the samples at 4°C overnight, wells were washed twice with TBS-T (50 mM Tris-HCl, pH 7.8, 150 mM NaCl, 1 mM MgCl_2 _and 0.05% Tween 80). The surface was blocked with 3% powdered skim milk in TBS for 1.5 h at room temperature followed by three steps of washing with TBS-T. Samples were then treated with the primary antibody for 1.5 h at room temperature, using a 1:1500 dilution of the antiserum pAK MspA#813 [8] in TBS. The wells were washed five times with TBS-T and were incubated for 1 h at room temperature with a 1:7500 dilution of Peroxidase-conjugated AffiniPure F (ab') 2 Fragment Goat Anti-Rabbit IgG (H+L) (Jackson Immuno Research, Soham, UK) in TBS. After five steps of washing, the reaction was performed using the SureBlue™ TMB Microwell Peroxidase Substrate (KPL, Gaithersburg, MD, USA) according to the instructions of the manufacturer. Absorption at 450 nm was measured with the microplate reader SPECTRA Fluor (TECAN, Crailsheim, Germany).

### Detection of PorMs at the surface of mycobacteria by means of quantitative microwell immunoassays

40 ml of mycobacterial culture was harvested at OD_600 _of 0.8, washed with PBS-T and the pellet was resuspended in 1 ml PBS-T. 200 μl aliquots were then incubated for 30 min on ice with 1 μl of antiserum (pAK MspA#813); for detection of background pre-immune serum was given to the samples. Afterwards 1 ml PBS-T was given to each sample; mycobacteria were harvested by centrifugation and washed once with PBS-T. Pellets were resuspended in 100 μl of PBS-T, 1 μl of the secondary Peroxidase-conjugated AffiniPure F (ab') 2 Fragment Goat Anti-Rabbit IgG (H+L) (Jackson Immuno Research) was added to each sample and bacilli were incubated on ice for 30 min. After addition of 1 ml PBS-T, mycobacteria were pelleted by centrifugation and were washed once with PBS-T. Pellets were then resuspended in 500 μl of PBS-T, and 100 μl of dilutions thereof were transferred to wells of a Nunc-Immuno Polysorp Module (Nalgene Nunc International). After addition of 100 μl SureBlue™ TMB Microwell Peroxidase Substrate (KPL) and stopping the reaction by addition of 50 μl 1 M HCl, the reaction was detected by the reader SPECTRAFluor (TECAN).

### Complementation of the porin-deficient mutant strain *M. smegmatis *ML10 with *porM1 *and *porM2*

The ability of *porM1 *and *porM2 *to complement the growth defect of *M. smegmatis *ML10 (*ΔmspA; ΔmspC*) [4] was examined by electroporation with the plasmids pSRa102, pSRa104, pSSa100 (Table 4) as well as the control pMV306. 750 ng of each plasmid was electroporated into *M. smegmatis *ML10 as described in Sharbati-Tehrani et al. [13]. After electroporation the cells were diluted and plated onto Mycobacteria 7H11 agar supplemented with kanamycin (25 μg/ml) for the assessment of growth after four days and for the quantification of growth by cfu counting during four days.

**Table 4:** Plasmids used in this work.

Plasmids	Characteristics	Reference
pIV2	cloning vector with an origin of replication functional in *Enterobacteriacea *and a kanamycin resistance gene	[39]

pLitmus38	cloning vector with the origin of replication from pUC, an ampicillin resitance gene and the *lacZ*' gene for blue/white selection	New England Biolabs

pMV306	cloning vector replicating in *E. coli *with the kanamycin resistance gene *aph *from transposon Tn903 and the gene for the integrase and the attP site of phage L5 for integration into the mycobacterial genome	[40]

pMV261	*Mycobacterium*/*E. coli *shuttle vector with the kanamycin resistance gene *aph *from transposon Tn903 and the promoter from the *hsp60 *gene from *M. tuberculosis*	[40]

pSHKLx1	*Mycobacterium*/*E. coli *shuttle vector with a kanamycin resistance gene from Tn5, a hygromycin resistance gene and the promoter from the *hsp60 *gene from *M. tuberculosis*	[43]

pSSa100	pMV306 with a 3429 bp genomic DNA fragment from *M. smegmatis *SMR5 carrying *mspA*	[13]

pSSp107, pSSp108	pIV2 with a 2895 bp genomic DNA fragment from *M. fortuitum *10860/03 carrying the *porM1 *gene	This study

pSRb101	pMV261 carrying the *porM1 *gene from *M. fortuitum *10860/03	This study

pSRb103	pMV261 carrying the *porM2 *gene from *M. fortuitum *10851/03	This study

pSRa102	pMV306 carrying the *porM1 *gene from *M. fortuitum *10860/03	This study

pSRa104	pMV306 carrying the *porM2 *gene from *M. fortuitum *10851/03	This study

pSRr106	pSHKLx1 carrying a 100 bp genomic DNA fragment from *M. fortuitum *10860/03 containing the beginning of the *porM1 *gene with the SD-sequence in antisense-orientation with respect to the *hsp60 *promoter	This study

### Knock-down of *porM *expression and over-expression of *porM1 *or *porM2 *in *M. fortuitum*

In order to accomplish a simultanous knock-down of *porM1 *and *porM2*, we generated a plasmid containing a transcriptional fusion of the *hsp60 *promoter with the 5' region of *porM *genes. The primers porM1-as-1 and porM1-as-2 were used to amplify a 100 bp PCR amplicon covering the 5' region of *porM1 *including the Shine-Dalgarno Sequence. The PCR product was cloned into the *BamHI *site of pSHKLx1 [43], and recombinant plasmids containing the insert in antisense orientation with respect to the *hsp60 *promoter were identified by sequencing. Afterwards, the selected recombinant plasmid pSRr106 was introduced into *M. fortuitum *by electroporation. The knock-down efficiency of the introduced antisense RNA was analysed at transcriptional level. For this purpose, RNA was isolated from *M. fortuitum *strains containing either pSRr106 or pSHKLx1, and porin expression was measured by SYBR Green qRT-PCR as described above.

Over-expression of *porM1 *or *porM2*, was achieved by introducing plasmids pSRb101 or pSRb103, respectively, into *M. fortuitum*.

## Authors' contributions

SS conceived the study, performed experiments and analyses and wrote and edited the manuscript. KS performed experiments, supervised the work of SR, HW and RA and designed their experiments. SR, HW, RA, VT and RK performed experiments and analyses. AL contributed to the experimental designs, writing and composition of the manuscript. All authors read and approved the final manuscript.

## Supplementary Material

Additional file 1 **Growth rate of the *M. fortuitum *strains 10851/03, 10860/03 and DSM 46621.** Logarithmic display of the growth curves shown in Figure 1. The growth rate of the strains was measured by quantification of the ATP-content [displayed as relative light units (RLU)] in broth cultures. Click here for file

Additional file 2 **Detection of the PorM spot on the 2D-PAGE by Western Blot analysis.** Detection was performed using the porin-specific antiserum pAK MspA#813 on the blotted 2D-PAGE shown in Figure 5A. Only one protein spot was identified possessing an apparent molecular mass of approximately 120 kDa and an apparent pI of about 4. The arrow indicates the identified spot. Click here for file

Additional file 3 **Western Blot analysis of PorMs in *M. fortuitum*.** Porin expression in members of the *M. fortuitum*-group was studied by Western blotting. 10–30 μg of protein extracted with nOPOE was separated by 1D-SDS-PAGE and detected by the antiserum pAK MspA#813. Lanes 1–4: 1, *M. smegmatis *SMR5 (10 μg); 2, *M. fortuitum *DSM 466211 (30 μg); 3, *M. fortuitum *10851/03 (30 μg); 4, *M. fortuitum *10860/03 (30 μg). Click here for file

Additional file 4 **Detection of PorMs on the surface of *M. fortuitum*.** Detection was performed using the porin-specific antiserum pAK MspA#813 in quantitative microwell immunoassays. Each column represents the mean (± SD) of 8 measurements. Asterisks indicate significant differences between the samples, which were treated with pAK MspA#813 and backgrounds according to the paired Student's t-test (P < 0.001). Click here for file

Additional file 5 **Knock-down of porins in *M. fortuitum *10860/03 by means of anti-sense technology using the plasmid pSRr106. **The amount of *porM1*/*porM2 *mRNA was quantified by means of qRT-PCR and was normalised with 16S rRNA. Compared to the reference strain *M. fortuitum *10860/03 (pSHKLx1) the amount of *porM *mRNA in the down-regulated strain 10860/03 (pSRr106) was reduced by about 75%. Click here for file
