# Supplementary figures and images for: Characterisation of porin genes from Mycobacterium fortuitum and their impact on growth
Source: BMC Microbiol. 2009 Feb 9;9:31. doi: 10.1186/1471-2180-9-31 (PMC2651896; doi:10.1186/1471-2180-9-31)

## Slide 1
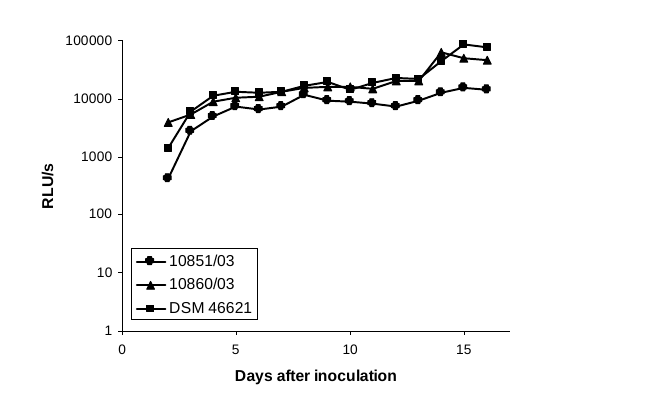

Supplement: Additional file 1 — Growth rate of the M. fortuitum strains 10851/03, 10860/03 and DSM 46621. Logarithmic display of the growth curves shown in Figure 1. The growth rate of the strains was measured by quantification of the ATP-content [displayed as relative light units (RLU)] in broth cultures. [file 1471-2180-9-31-S1.ppt]

## Slide 1
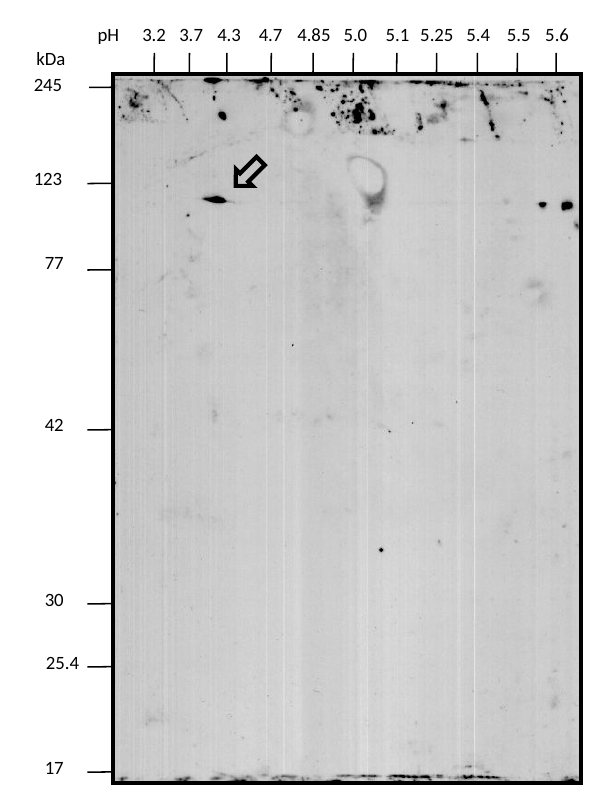

pH
3.2
3.7
4.3
4.7
4.85
5.0
5.1
5.25
5.4
5.5
5.6
kDa
245
123
77
42
30
25.4
17

Supplement: Additional file 2 — Detection of the PorM spot on the 2D-PAGE by Western Blot analysis. Detection was performed using the porin-specific antiserum pAK MspA#813 on the blotted 2D-PAGE shown in Figure 5A. Only one protein spot was identified possessing an apparent molecular mass of approximately 120 kDa and an apparent pI of about 4. The arrow indicates the identified spot. [file 1471-2180-9-31-S2.ppt]

## Slide 1
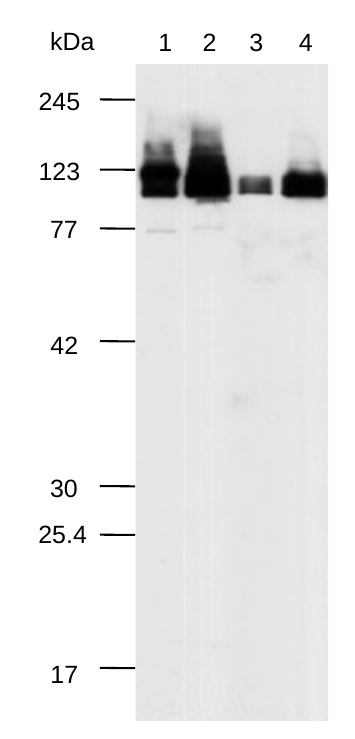

kDa
1
2
3
4
245
123
77
42
30
25.4
17

Supplement: Additional file 3 — Western Blot analysis of PorMs in M. fortuitum. Porin expression in members of the M. fortuitum-group was studied by Western blotting. 10–30 μg of protein extracted with nOPOE was separated by 1D-SDS-PAGE and detected by the antiserum pAK MspA#813. Lanes 1–4: 1, M. smegmatis SMR5 (10 μg); 2, M. fortuitum DSM 466211 (30 μg); 3, M. fortuitum 10851/03 (30 μg); 4, M. fortuitum 10860/03 (30 μg). [file 1471-2180-9-31-S3.ppt]

## Slide 1
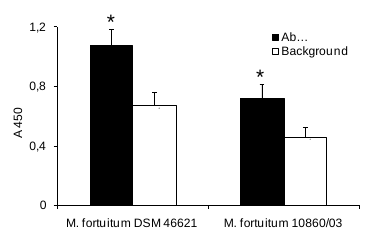

*
*

Supplement: Additional file 4 — Detection of PorMs on the surface of M. fortuitum. Detection was performed using the porin-specific antiserum pAK MspA#813 in quantitative microwell immunoassays. Each column represents the mean (± SD) of 8 measurements. Asterisks indicate significant differences between the samples, which were treated with pAK MspA#813 and backgrounds according to the paired Student's t-test (P < 0.001). [file 1471-2180-9-31-S4.ppt]

## Slide 1
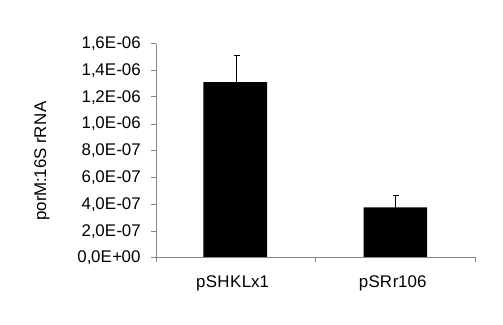

Supplement: Additional file 5 — Knock-down of porins in M. fortuitum 10860/03 by means of anti-sense technology using the plasmid pSRr106. The amount of porM1/porM2 mRNA was quantified by means of qRT-PCR and was normalised with 16S rRNA. Compared to the reference strain M. fortuitum 10860/03 (pSHKLx1) the amount of porM mRNA in the down-regulated strain 10860/03 (pSRr106) was reduced by about 75%. [file 1471-2180-9-31-S5.ppt]
